# Improved light extraction of InGaN/GaN blue LEDs by GaOOH NRAs using a thin ATO seed layer

**DOI:** 10.1186/1556-276X-7-458

**Published:** 2012-08-16

**Authors:** Hee Kwan Lee, Dong Hyuk Joo, Myung Sub Kim, Jae Su Yu

**Affiliations:** 1 Department of Electronics and Radio Engineering, Kyung Hee University, 1 Seocheon-dong, Giheung-gu, Yongin-si, Gyeonggi-do 446-701, Republic of Korea

**Keywords:** Light extraction, Light-emitting diodes, Gallium oxide hydroxide, Nanorod arrays, Electrochemical deposition

## Abstract

We investigated the effect of gallium oxide hydroxide (GaOOH) nanorod arrays (NRAs) on the light extraction of InGaN/GaN multiple quantum well blue light-emitting diodes (LEDs). GaOOH NRAs were prepared on an indium tin oxide electrode (ITO) layer of LEDs by electrochemical deposition method. The GaOOH NRAs with preferred orientations were grown on the ITO surface by sputtering a thin antimony-doped tin oxide seed layer, which enhances heterogeneous reactions. Surface density and coverage were also efficiently controlled by the different growth voltages. For LEDs with GaOOH NRAs grown at −2 V, the light output power was increased by 22% without suffering from any serious electrical degradation and wavelength shift as compared with conventional LEDs.

## Background

There has been growing interest in gallium nitride (GaN)-based light-emitting diodes (LEDs) as the most promising light source for automotive lightings, indoor/outdoor lighting products, backlight units for liquid crystal displays, and solid-state lightings [1]. However, the light generated in the active region of LEDs undergoes Fresnel reflection loss and a narrow escape cone due to high refractive index contrast at the GaN/air or indium tin oxide (ITO)/air interface when it is emitted out from the device. To solve these problems, various techniques including surface texturing/roughening and incorporation of photonic crystal structures, subwavelength structures, ZnO nanorods have been developed for GaN-based LEDs [2-7]. However, some methods require complex process steps and cause plasma-induced damage [2,7].

Gallium oxide hydroxide (GaOOH) is an important starting material for the synthesis of gallium oxide (Ga_2_O_3_) and gallium nitride (GaN) by simple heat treatments [8,9]. In recent years, extensive attention has been paid to the shape- and size-controlled synthesis of GaOOH nanostructures for device applications in optoelectronics and photonics [10-13]. GaOOH exhibits a high transparency in the ultraviolet and visible wavelength ranges, and its refractive index is lower than that of Ga_2_O_3_ of 1.8 to 1.9 because the hydroxide materials are less dense [11,12]. Thus, controllable growth in GaOOH nanorod arrays (NRAs) can efficiently provide a graded refractive index profile for GaN-based LEDs. GaOOH or Ga_2_O_3_ would also act as an efficient surface passivation layer [13]. Several growth methods, such as sol–gel process, sonochemical reaction, and hydrothermal synthesis, have been employed to synthesize the rodlike GaOOH structures [8,14,15]. However, the rod-shaped GaOOH in powder form, made by homogeneous nucleation from chemical solutions, was usually prepared. For more efficient device applications, it is essential to grow vertically aligned NRAs on substrates. It was found that the vertically aligned GaOOH NRAs can be grown on rigid substrates by chemical solution deposition (CSD) using thin metal oxide (SnO_2_ and MgO) seed layer [16]. These materials indicate the low lattice mismatch with GaOOH for *a*-axis (*a*_GaOOH_ = 4.58 Å, *a*_SnO2_ = 4.738 Å, *a*_MgO_ = 4.212 Å). However, the CSD process takes a long growth time, and it is not easy to control the growth process. Meanwhile, electrochemical deposition (ED) process is a simple, cost-effective, low-temperature, and fast growth method. In this work, we fabricated GaN-based blue LEDs with electrochemically grown GaOOH NRAs to enhance the light extraction efficiency. The GaOOH NRAs were successfully synthesized on the ITO surface of LEDs by the use of a thin antimony (Sb)-doped SnO_2_ (ATO) seed layer, which is one of the transparent conducting oxide materials and indicates a refractive index of 1.8 to 2 in the visible wavelength range [17]. Compared with conventional LEDs, the optical and electrical characteristics of LEDs with GaOOH NRAs were investigated.

## Methods

GaN-based blue LED structures were grown on sapphire (Al_2_O_3_) substrate by metal organic chemical vapor deposition. The epilayer structure consisted of a 3.5-μm-thick undoped GaN layer, a 4-μm-thick Si-doped n-type GaN layer, five pairs of InGaN/GaN multiple quantum wells (MQWs), and a 210-nm-thick Mg-doped p-type GaN layer. The LEDs were fabricated with a mesa size of 400 × 450 μm^2^ in a lateral device using conventional fabrication processes. After mesa etching process, a 200-nm-thick ITO layer was deposited on the p-type GaN layer using an e-beam evaporator, and it was annealed at 600°C for 60 s in air ambient. The Cr/Au (10/500 nm) layers were used as the *p*- and *n*-metal electrodes. In the fabricated LEDs, GaOOH NRAs were synthesized on the ITO surface by ED method.

**Figure 1:**
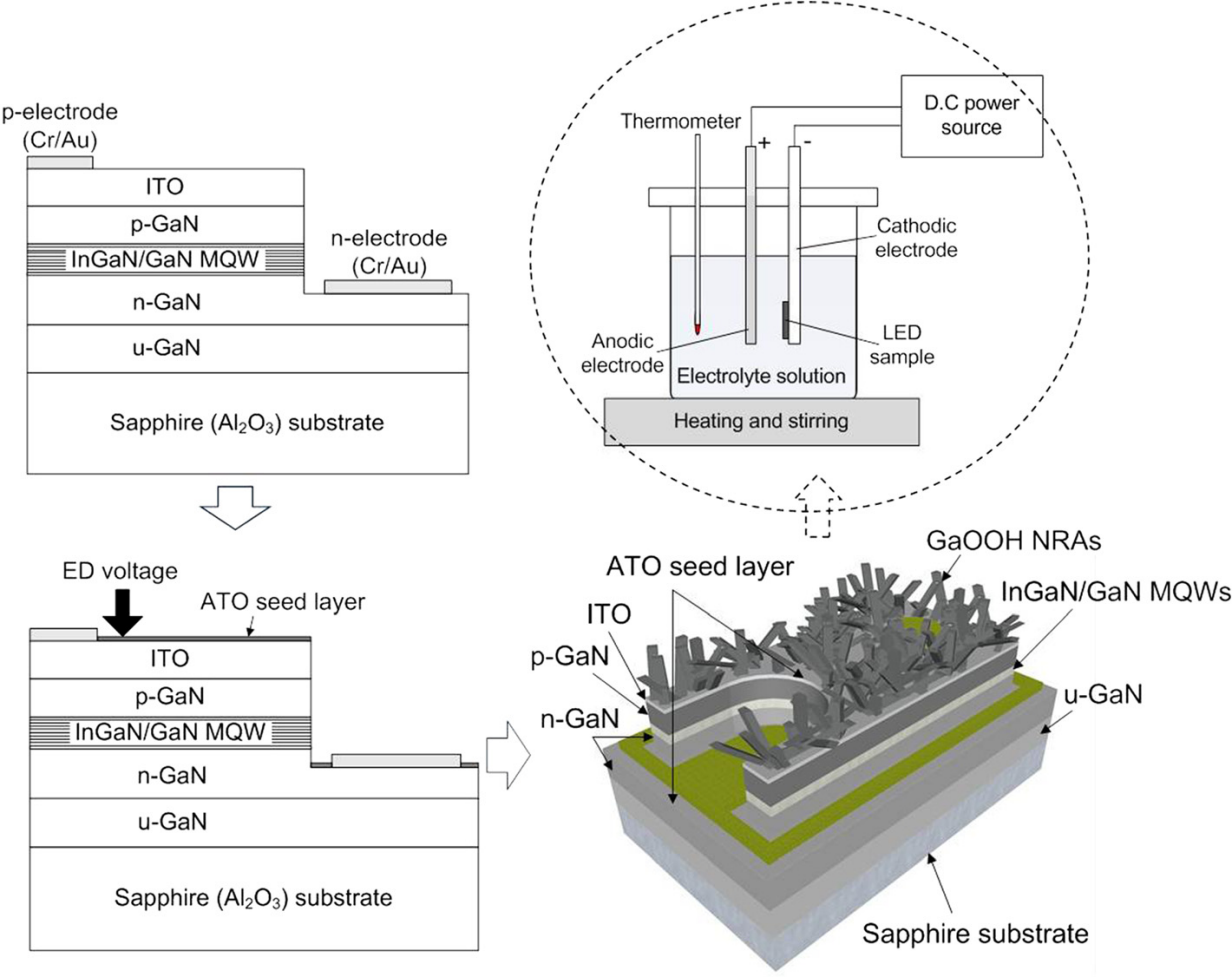
Fabrication procedure’s schematic diagram for InGaN/GaN MQW blue LEDs with GaOOH NRAs by ED method.

Figure 1 shows the schematic diagram of the fabrication procedure for InGaN/GaN MQW blue LEDs with GaOOH NRAs by ED method. To grow the GaOOH NRAs, a thin ATO seed layer was deposited on the ITO surface of LEDs using RF magnetron sputtering system at room temperature without masking the electrodes for a simple fabrication process. The LED samples were mounted on the sample holder. The sample holder and platinum (Pt) electrode were then immediately immersed into the chemical solution, consisting of gallium nitrate (5 mM, Ga(NO_3_)_3_·*x*H_2_O), ammonium nitrate (5 mM, NH_4_NO_3_), and deionized (DI) water. The LED sample and the Pt electrode were used as cathodic and anodic electrodes, respectively. The distance between the electrodes was about 1 cm. For 100 min of growth time, a constant cathodic voltage was applied to the top surface (i.e., *p*-electrode and ITO surface) of LED samples versus the Pt electrode, and the growth temperature was fixed at 80 °C. Also, the working solution was stirred at 80 rpm for reaction time. When the growth process finished, the samples were immediately taken out from the solution and rinsed in flowing DI water several times to remove any unwanted residues and then dried with a flow of pure N_2_ gas. The morphologies of electrodeposited GaOOH NRAs were characterized by field emission scanning electron microscopy (FE-SEM). The crystal structure was analyzed by X-ray diffraction (XRD) with monochromated CuK*α* irradiation (*λ* = 1.54056 Å). The optical and electrical properties of the fabricated LEDs were analyzed, and the electroluminescence (EL) was also measured at room temperature.

## Results and discussion

**Figure 2:**
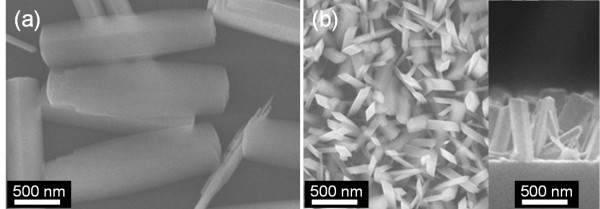
**SEM images of GaOOH rods synthesized on Si substrates.** (**a**) Without and (**b**) with an ATO seed layer under a cathodic voltage of −2 V at 80°C.

Figure 2a,b shows the top-view SEM images of GaOOH rods synthesized on Si substrates without and with a thin sputtered ATO seed layer under a cathodic voltage of −2 V at 80°C. In general, GaOOH precipitates are easily formed in aqueous solutions by the following reaction [8]:

$${\text{Ga}}^{3+}+2{\text{H}}_{2}\text{O}\to \text{GaOOH}+3{\text{H}}^{+}$$

At high temperature, the OH^−^ ion is sufficiently generated in water, and GaOOH can be easily synthesized by homogeneous nucleation [15]. In the ED method, similarly, the growth processes were carried out in aqueous gallium nitrate solution at 80°C. Thus, the white-colored GaOOH precipitates were naturally formed in the chemical solution during the reaction time. For ED process, the growth of GaOOH rods is mainly based on the generation of OH^−^ ions at the cathode electrode. When the certain voltage is applied, the OH^−^ ions are produced near the cathode electrode by electrochemical reduction of precursors such as NO_3_^−^ and O_2_ in aqueous gallium nitrate solution [18]. The Ga^3+^ ions then react with the OH^−^ ions, and GaOOH rods are formed. First, the influence of the seed layer on the growth property of GaOOH rods was investigated. In the case of the growth process without the seed layer, only a few GaOOH rods were observed on the substrate, and the rods were located parallel to the surface, as can be seen in Figure 2a. This means that the GaOOH rods were synthesized through homogeneous nucleation without the influence of a substrate. The synthesized GaOOH rods indicated the rhombus-shaped structures with an average rod length of approximately 1.9 μm and lateral dimensions of 200 to 600 nm. Under a cathodic voltage, the heterogeneous nucleation was enhanced by depositing a thin ATO seed layer on Si substrate. As shown in Figure 2b, the GaOOH NRAs were vertically and uniformly synthesized on the ATO/Si surface. The morphology of GaOOH NRAs indicated a rhombus-shaped structure. The GaOOH NRAs had an average nanorod length of approximately 520 nm, and the lateral dimension was roughly varied from 50 to 220 nm.

**Figure 3:**
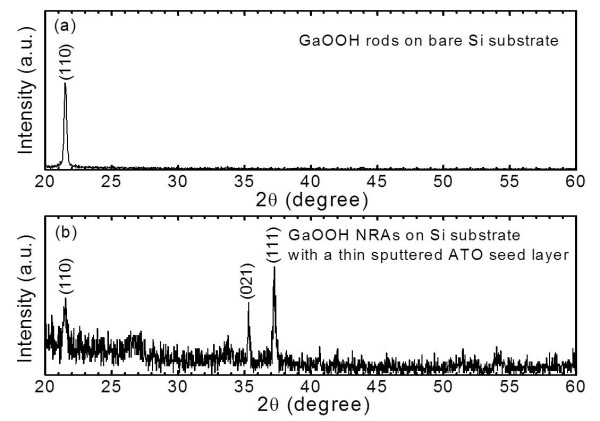
**XRD patterns of the GaOOH NRAs grown on Si substrates.** (**a**) Without and (**b**) with an ATO seed layer under a cathodic voltage of −2 V at 80°C.

Figure 3 shows the 2*θ* scan XRD patterns of the GaOOH nanorods grown on Si substrates without and with a thin sputtered ATO seed layer under a cathodic voltage of −2 V at 80°C. All diffraction peaks were indexed to the orthorhombic α-GaOOH phase without any impurity phase. For bare Si substrate, the strong diffraction peak of (110) was only observed because the GaOOH rods lie parallel to the substrate, as shown in Figure 2a. In the case of GaOOH NRAs with the ATO seed layer, the diffraction peaks were assigned to (110), (021), and (111) plane orientations. The diffraction intensity of (111) peak was dominant. These results show that GaOOH NRAs can be efficiently formed on the substrate with a thin ATO seed layer by the ED method. Also, the ATO seed layer plays an important role in controlling the oriented growth of GaOOH NRAs by enhancing the heterogeneous nucleation on the substrate.

**Figure 4:**
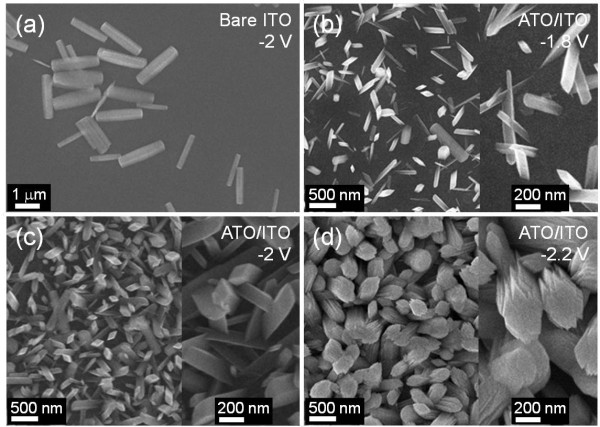
**SEM images of GaOOH rods.** These rods are grown on ITO electrode of LEDs with and without ATO seed layer under different cathodic voltages for 100 min at 80°C. (**a**) Bare ITO, −2 V; (**b**) ATO/ITO, −1.8 V; (**c**) ATO/ITO, −2 V; and (**d**) ATO/ITO, −2.2 V.

In order to investigate the effect of cathodic voltage on the growth properties, the GaOOH NRAs were synthesized on the ITO electrode of LEDs with/without the ATO seed layer under various cathodic voltages for 100 min. The cathodic voltage was changed from −1.8 to −2.2 V. Figure 4a shows the top-view SEM image of GaOOH rods grown on bare ITO electrode under a cathodic voltage of −2 V. The precipitation behavior on bare ITO electrode was similar to that formed on bare Si substrate. Only a few submicrometer-sized GaOOH rods lying parallel to the ITO electrode were observed, which indicates the homogeneous nucleation of GaOOH. In the case of the growth process with the ATO seed layer, the GaOOH NRAs were synthesized by the heterogeneous nucleation on the ATO/ITO surface. For all samples, the GaOOH NRAs were grown with preferred orientations on the ITO electrode as shown in Figure 4b,c,d. At −1.8 V, the GaOOH NRAs were sparsely synthesized. Additionally, the homogeneous nucleation of GaOOH rods was partially observed. As the cathodic voltage increased, the growth rate became higher, and the surface coverage was increased. At −2 V, the GaOOH NRAs were more densely and uniformly synthesized on the ATO/ITO surface. The average nanorod length was approximately 520 nm, and the lateral dimension became larger than that of GaOOH nanorods grown at −1.8 V. But most of the GaOOH nanorods were smaller than 260 nm. For an excessive cathodic voltage of −2.2 V, the ATO/ITO surface was almost wholly covered by large GaOOH NRAs with lateral dimensions above 300 nm. Also, the average nanorod length was increased by approximately 800 nm. The different cathodic voltage provides different amount of electrons for the growth reaction, and the increased voltage promotes the generation of OH^−^ ions by electrochemical reduction of NO_3_^−^ and dissolved O_2_ near the cathodic electrode. Thus, the more GaOOH nuclei are formed on the ATO/ITO surface, and the surface density is increased. Also, the growth reactions are accelerated.

**Figure 5:**
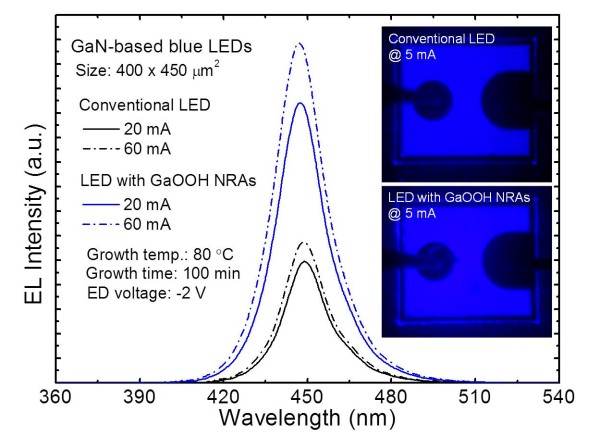
**Room-temperature EL spectra of LEDs with and without GaOOH NRAs at various injection currents.** The inset shows the illumination images of the fabricated LEDs with/without GaOOH NRAs at an injection current of 5 mA.

Figure 5 shows the room-temperature EL spectra of InGaN/GaN MQW blue LEDs with and without electrochemically grown oriented GaOOH NRAs on ITO surface using a thin sputtered ATO seed layer. The GaOOH NRAs were synthesized under a cathodic voltage of −2 V for 100 min. The EL peak wavelength was not shifted after the growth process of GaOOH NRAs. For all LEDs with/without GaOOH NRAs, the emission peak wavelength was observed at *λ* = 448.81 and 446.85 nm for 20 and 60 mA, respectively. This shows that the formation of GaOOH NRAs on GaN-based LEDs does not induce any radiative defects or surface damages. Furthermore, the EL intensity of LED with GaOOH NRAs was improved compared with the conventional LED. The inset shows the illumination image of the fabricated InGaN/GaN MQW LEDs with/without oriented GaOOH NRAs at an injection current of 5 mA. The LED with oriented GaOOH NRAs was brighter than the conventional LED. From these results, the light output power of GaN-based LEDs can be efficiently improved by the electrodeposited GaOOH NRAs.

**Figure 6:**
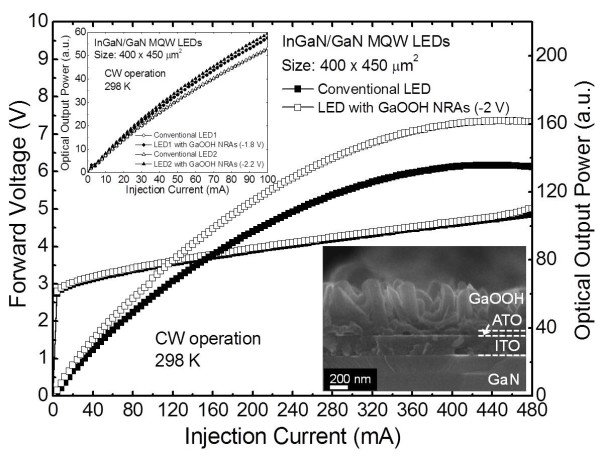
**Measured *L*-*I*-*V *curves of LEDs with and without GaOOH NRAs.** They are grown at −2 V in CW operation at 298 K. The inset shows the *L*-*I* curves (top) of the LEDs with and without GaOOH NRAs grown at different cathodic voltages of −1.8 and −2.2 V and the cross-sectional SEM image (bottom) of GaOOH NRAs grown on the ATO/ITO surface of the LED at −2 V.

Figure 6 shows the measured light-current–voltage (*L*-*I*-*V*) curves of the fabricated InGaN/GaN MQW blue LEDs with and without oriented GaOOH NRAs grown under a cathodic voltage of −2 V. The inset shows the measured *L*-*I* curves of the fabricated InGaN/GaN MQW blue LEDs with and without oriented GaOOH NRAs grown at different cathodic voltages. The FE-SEM image of LED with GaOOH NRAs on ATO/ITO films is also shown in the inset of Figure 6. The *L*-*I*-*V* measurements were carried out using a probe station system. The samples were loaded on the copper mount with a thermistor to control a constant temperature of 298 K. The device characteristics were not affected by a thin ATO seed layer. After the formation of GaOOH NRAs, the forward voltages were 2.98 and 3.03 V at 20 mA for the convention LED and the LED with GaOOH NRAs, respectively. The very slight increment may be caused by the GaOOH NRAs grown on the *p*-electrode because it is not masked for a simple fabrication process. However, there was no distinct electrical degradation. This indicates that the ATO layer only acts as a seed layer to grow the oriented GaOOH NRAs without sacrificing the electrical and optical properties. Compared with conventional LED, the light output power was increased by 22% for LEDs with GaOOH NRAs at an injection current of 20 mA. The integrated GaOOH NRAs may offer the graded refractive index between the ITO electrode and air, as well as the textured surface. Thus, the internal reflection is efficiently reduced, and more photons can escape from the GaOOH NRAs/ITO surface. Meanwhile, the saturation behavior of the maximum output power was observed at similar current injection levels for both samples. For conventional LED, the output power was saturated at 468 mA, and it was slightly increased to 470 mA for the LED with GaOOH NRAs. As shown in the inset of Figure 6, the maximum limitation in light output power was also investigated for different surface coverage and rod lengths. For LEDs with GaOOH NRAs grown at −1.8 and −2.2 V, the improvement in light output power was 8 and 12%, respectively. As the cathodic voltage increases, the ITO surface is wholly covered by the GaOOH NRAs with higher surface coverage and longer rod lengths, and it eventually indicates the close-packed morphology. The close-packed morphology of GaOOH NRAs may hinder effective light scattering, and the longer rod lengths lead to high light absorption loss. Consequently, the improvement in light extraction efficiency of GaN-based LEDs can be efficiently obtained by integrating controllably grown GaOOH NRAs under optimization condition.

## Conclusions

GaN-based LEDs with GaOOH NRAs on ITO electrode were fabricated by the ED method. The oriented growth of GaOOH NRAs on the ITO electrode was successfully obtained by the use of a thin sputtered ATO seed layer. The surface coverage and density of GaOOH NRAs were controlled by the cathodic voltage. In comparison with the conventional LEDs, the enhancement of 22% in light output power was achieved by the incorporation of GaOOH NRAs without any radiative defects. The electrical properties also were not distinctly affected by the growth process in GaOOH NRAs.

## Competing interests

The authors declare that they do not have competing interests.

## Authors’ contributions

HKL proposed the original idea, carried out most of the experimental works associated with fabrication and characterization of samples, analyzed the results, and prepared the manuscript. DHJ and MSK helped the experimental works and characterization of samples. JSY developed the conceptual framework, supervised the whole work, and finalized the manuscript. All authors read and approved the final manuscript.
